# Specific phobia and medical aptitude for work : Case report

**DOI:** 10.1192/j.eurpsy.2023.1424

**Published:** 2023-07-19

**Authors:** M. N. Fendri, N. Kammoun, A. Wiem, H. Anwar, S. Ernez, F. Sonia, M. Bani, N. Habib

**Affiliations:** Médecine du travail, Institut de Santé et Sécurité au Travail; 2 Médecine du travail, Hopital Charles Nicolle, Tunis, Tunisia

## Abstract

**Introduction:**

Specific phobia is an excessive and unreasonable fear of an object or situation that does not represent a real danger. This disorder is widespread among the population. The suffering from the feared situation disturbs the individual’s habits and professional activities.

**Objectives:**

We report the case of a driving phobia in a professional driver.

**Methods:**

Case report

**Results:**

The man was 32 years old, a smoker at 5 PA. He had no family or personal psychiatric history. He has been a Dumper machine driver in a phosphate extraction company since 2011. He presented to our institute for a fit-to-work assessment. The history of the disease dates to 2019, the patient had witnessed a work accident that caused the death of his colleague (engine driver). Since this accident, he had a state of anxiety associated with tachycardia, a feeling of suffocation, excessive sweating and headaches. This symptomatology occurred suddenly while driving and prevented the patient from performing his professional task. At the psychiatric interview, the patient had coherent and dynamic speech without psychomotor slowing. The rest of the clinical examination was normal. The patient had been referred to a psychiatrist. The diagnosis of a specific phobia had been retained. Given the anamnestic and clinical data and the opinion of a medical specialist, the patient had been placed on temporary incapacity for the driving position. A reassessment of his medical fitness for the position of the driver will be made after the end of the psychiatric intake.

**Conclusions:**

Professional conduct is a complex task that requires the integrity of physical and mental abilities. The assessment of medical fitness for this position is essential for road safety. However, it can sometimes be difficult, especially in the face of psychiatric pathologies.

**Disclosure of Interest:**

None Declared

